# Electrical Charge Coupling Dominates the Hysteresis Effect of Halide Perovskite Devices

**DOI:** 10.1021/acs.jpclett.2c03812

**Published:** 2023-01-24

**Authors:** Juan Bisquert

**Affiliations:** Institute of Advanced Materials (INAM), Universitat Jaume I, 12006 Castelló, Spain

## Abstract

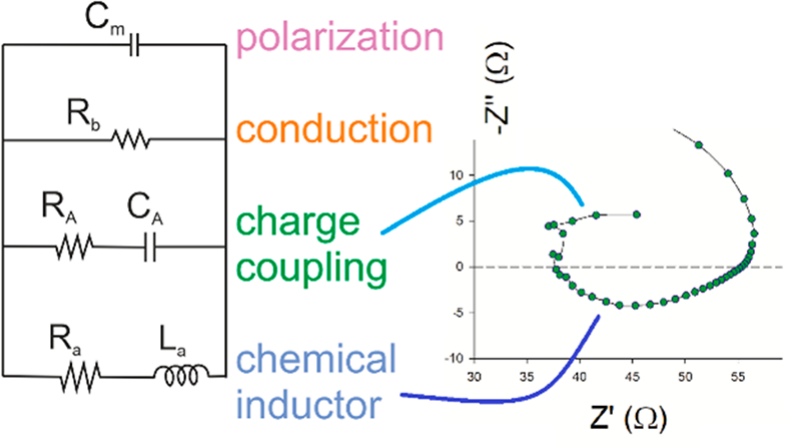

Hysteresis effects in ionic-electronic devices are a valuable resource for the development of switching memory devices that can be used in information storage and brain-like computation. Halide perovskite devices show frequent hysteresis in current–voltage curves that can be harnessed to build effective memristors. These phenomena can be often described by a set of highly nonlinear differential equations that involve current, voltage, and internal state variables, in the style of the famous Hodgkin–Huxley model that accounts for the initiation and temporal response of action potentials in biological neurons. Here we extend the neuron-style models that lead to chemical inductors by introducing a capacitive coupling in the slow relaxation variable. The extended model is able to explain naturally previous observations concerning the transition from capacitor to inductor in impedance spectroscopy of MAPbBr solar cells and memristors in the dark. The model also generates new types of oscillating systems by the generation of a truly negative capacitance distinct from the usual inductive effect.

Many types of electronic, electrochemical, and biological systems show a memory effect in which the electrical response to an external stimulus depends on the past history of the sample. This feature can be a problem to be eliminated in electronic and energy conversion devices, as in the hysteresis in solar cells, or otherwise it may consist of a central part of the functionality. The latter is the case of biological neurons that transfer information to their neighbors by action potentials.^1−3^ The memory effect is also necessary in memristors that allow the storage of information by metastable modification of device conductivity.^4−8^ Either for better control, or suppression of the property, it is important to obtain a general perspective with quantitative models able to cover different experimental methods across several related fields.

Recently, the hysteresis in current voltage curves of photovoltaic halide perovskites has been the subject of many discussions.^9,10^ The hysteresis effect is generally attributed to the combination of ionic and electronic transport, and recombination and polarization phenomena.^11−14^ Dynamic hysteresis occurs in different types. The hysteresis loop in the dark can be clockwise in “regular hysteresis” or counterclockwise in “inverted hysteresis”.^15^ The problem is that many interpretations are based on apparently different models. A connection has been established between the type of hysteresis and the dominant features in the equivalent circuit of impedance spectroscopy.^16,17^ This is also a matter of general interest since the inductive hysteresis turns out to be related to the famous negative capacitance widely observed across different disciplines.^18^ It is suggested that a stabilized negative capacitance holds the key for future generations of low consumption microelectronics.^19,20^

We have developed unified explanations of these phenomena by means of a set of models^17,21−23^ that have a relatively simple structure and appear in many areas of research. The models are formed by a small set of highly nonlinear differential equations: a conduction equation and several delay equations, as described in detail below. Historically these models obtained celebrity by the Hodgkin–Huxley (HH) model of biological neurons.^1−3^ The model does not describe in detail every molecular aspect of the biological cells but accounts very successfully for the initiation and temporal response of action potentials and is henceforth a central piece of neuroscience.

The topic of this paper is to develop a generalization of “neuron-style” models by a coupling term not included in the original HH model. We therefore introduce a generalization of the previous set of models and analyze the new properties represented in small signal ac impedance spectroscopy. We discuss which model better fits the evidence of halide perovskite experiments on hysteresis, and finally we describe the general properties of bifurcation, stability, and the appearance of a negative capacitance.

Let us describe a conduction/polarization system using a set of nonlinear coupled dynamical equations
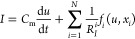
1
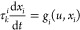
2

Equations 1–2 form a well established framework analysis of different classes of systems. Here *u* is the voltage, and *I* is the current across the device. The first equation indicates that the current *I* is composed of several parts. The initial term is a capacitive charging with capacitance *C*_m_, that exists in practically all systems, at least by the geometric capacitance effect, combined with possible electrode capacitances. The second term in eq 1 is a combination of conduction currents. Each mechanism is described by a conductivity function *f*_*i*_ (that may itself contain parallel pathways, see below) with resistance scale parameters *R*_I_^*i*^ and is furthermore affected by an additional internal variable denoted *x*_*i*_. This variable causes the memory effect by a slow recovery of relaxation time τ_*k*_^*i*^ in response to the changes, by a voltage-driven adaptation function *g*_*i*_ (*x*_*i*_,_*u*_), as indicated in eq 2.

There are a total of *N* internal variables in the model, according to the dynamic complexity of the system. In neuron models *C*_m_ is the capacitance of the membrane, and *x*_*i*_ are different variables controlling the voltage-gated conductivity across the ion channels. The FitzHugh–Nagumo,^24,25^ the Morris-,Lecar^26^ and the Wilson^27^ neuron models have all one variable, while the Hodgkin–Huxley model has a total of three slow variables.^1,2,28,29^ Additionally, we often find in neuron models^2^ a total decoupling of the conduction current into two parallel branches as follows^30^
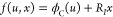
3

Here the first term is an instantaneous current ϕ_C_/*R*_I_ and the slow variable *x* is normally called an adaptation current.^3,31^

Equation 1 forms a generalization of traditional memristor models, that normally have only one internal variable,^7,32,33^ although a recent halide perovskite memristor model has two internal variables.^22^ Another important aspect of eqs 1–2 is stability and bifurcation. Recently we have summarized^21,30,34^ a large variety of examples of single *x*–variable models (two-dimensional coupled equations) of the type of eqs 1 and 2 that include periodic oscillations by intrinsic instability, as in spiking neurons^3,35^ and electrocatalytic reactions.^36,37^

In this Letter we explore an extension of eqs 1 and 2 for the single *x*–variable class of models and analyze the implications for halide perovskite solar cells and memristors and the broader consequences for conduction dynamical systems.

Based on the eqs 1–2, we propose the following extended model
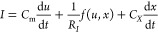
4
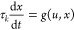
5

In eq 4, a derivative term *C*_*X*_ d*x*/d*t* has been added. This extension of eq 1 requires us to further define the physical interpretation of the variable *x*. Note that units of *x* and *C*_m_ must be adjusted so that *I* is in amperes, *u* is in volts, *C*_m_ is in farads, and *t*, τ_*k*_ in seconds.

Consider first that *x* is a current as it is typical in neuron models mentioned above as in eq 3.^2,3^ Then there is no d*x*/d*t* term needed, and we must set the constant *C*_*X*_ = 0.

Otherwise, let us consider that *x* is an internal charge variable *Q*_S_ or an internal voltage *V*_S_. The charging of this physical variable introduces a capacitance in addition to the passive capacitance *C*_m_, hence the need for the derivative term in (eq 4).^11,38^

In order to analyze the dynamical implications of the additional capacitive term, we calculate the small signal expansion, where the small perturbation quantities are denoted *ŷ*, and we apply the Laplace transform, d/d*t* → *s*, where *s* = *i*ω in terms of the angular frequency ω. We arrive at
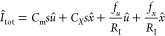
6
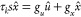
7

The impedance function takes the form
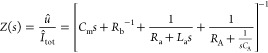
8

The circuit elements in eq 8 have the expressions that can be obtained from eqs 6–7
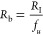
9
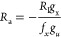
10
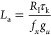
11
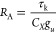
12
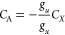
13

The correspondent equivalent circuit model is shown in Figure 1a. This generalized circuit contains the chemical inductor model (without the *C*_**X**_ term) characterized in recent publications.^21,23^ As shown previously in general terms, the model of eqs 1–2 always gives the (*R*_a_, *L*_a_) branch that forms a chemical inductor.^21^ We have the inductor time constant
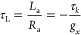
14

**Figure 1:**
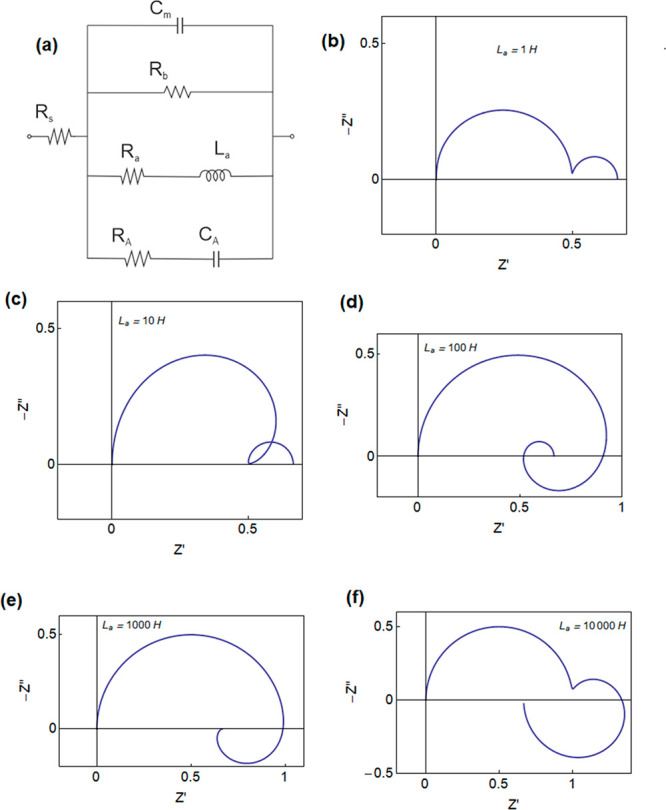
(a) Equivalent circuit model. (b–f) Set of impedance spectra generated for *C*_m_ = 10 F, *R*_a_ = 1 Ω, *R*_b_ = 2 Ω, *R*_d_ = 2 Ω, *C*_d_ = 1000 F, and *L*_a_ as indicated. *Z*′ and *Z″* are in units of Ω.

The new term in eq 4 creates the additional branch (*R*_A_, *C*_A_) shown in Figure 1a. This branch does not contribute to the total dc resistance that is given by
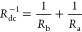
15

However, the (*R*_A_, *C*_A_) branch causes important effects at intermediate frequencies.^39^ If we calculate the associated time constant, we get
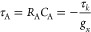
16

We obtain the remarkable property that the two branches of the slow mode have the same time constant
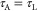
17

This property will be used below for the interpretation of experiments. It arises from the fact that the lower branches of Figure 1a originate from the same mechanism. We introduce also the time constant
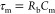
18

We conclude that current coupling and charge coupling in the slow variable of the model produces distinct effects in the impedance response, depending on the appearance of the branch (*R*_A_, *C*_A_) that satisfies the property (eq 17).

Figure 1 also shows a number of characteristic impedance spectra that are produced by the model of Figure 1a. The different shapes are obtained by the variation of the value of the inductor *L*_a_. For this element varying from small to large, we obtain a double arc structure (b), a loop at intermediate frequencies (c), a spiral (d), the characteristic loop in the positive *Z*″ (fourth quadrant of the complex plane) associated with the pure chemical inductor^21^ (e), and a double arc that finalizes in a chemical inductor (f). These spectra are highly characteristic of the impedance spectroscopy of halide perovskite solar cells and have been reported by many authors in recent years, as shown in several examples from the literature in Figure 2.

**Figure 2:**
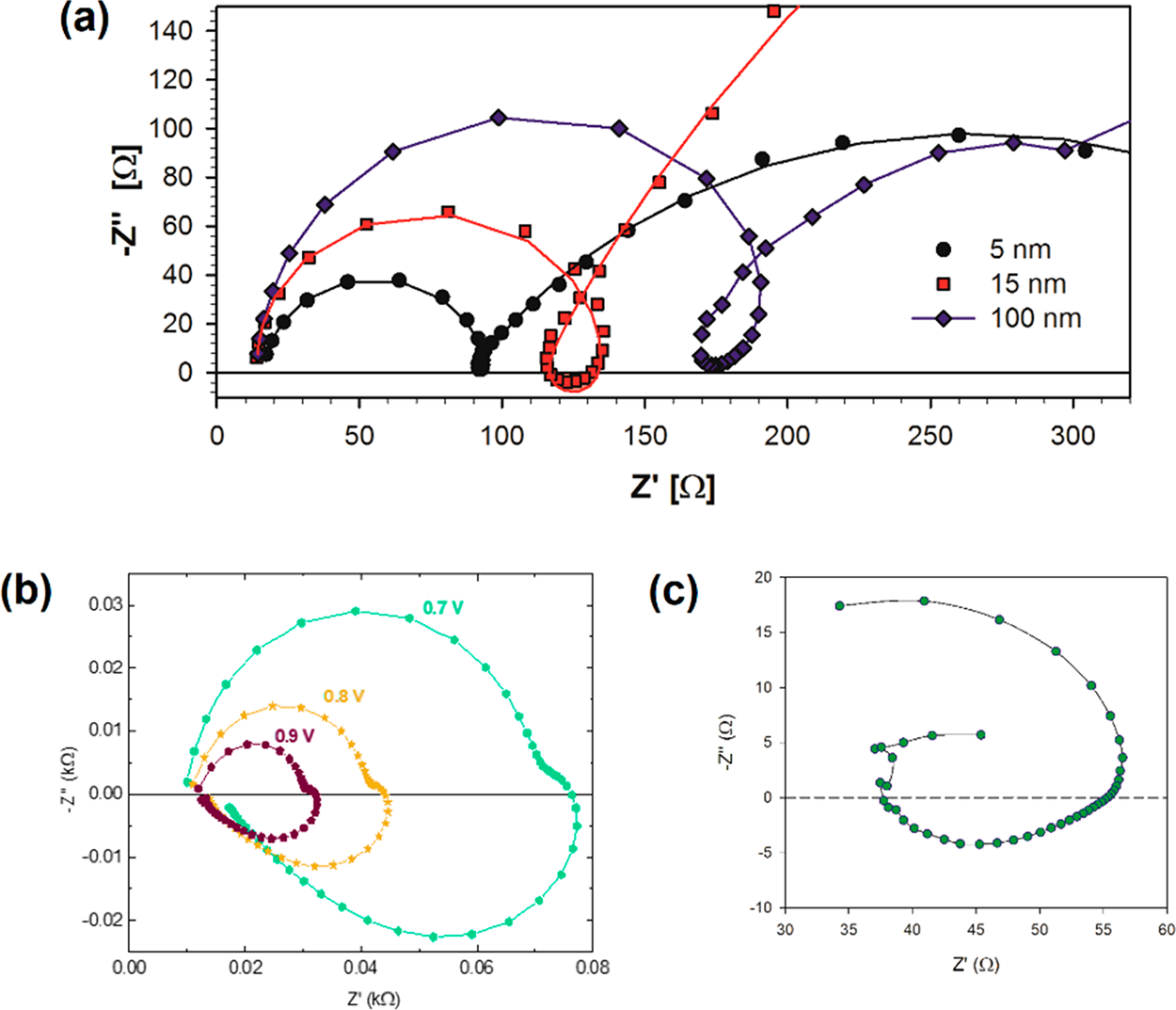
Complex impedance plot of (a) (FA_0.85_MA_0.15_Pb(I_0.85_Br_0.15_)_3_) photovoltaic devices containing electron transport layers of SnO_2_, of different thickness, measured at 1 sunlight intensity prepared with different electron transport layers. Reproduced from ref (40). Copyright 2016 American Chemical Society. (b) FTO/PEDOT:PSS/MAPI/Au memristor device at different applied dc voltage. Reproduced from ref (22). Copyright 2022 American Chemical Society. (c) MAPbBr solar cell under dark conditions at 1.6 V. Reproduced from ref (17). Copyright 2022 American Chemical Society.

The analysis of IS of halide perovskite devices is now widely reported.^41^ It is interesting to develop the interpretation by comparing different models toward the central goal of obtaining deep insight into the operation of the devices. Many authors have proposed interpretations of the hysteresis effects and negative capacitance effects by the ionic-electronic coupling that is broadly observed in halide perovskites.^11−14,18,41−44^ However, the detailed mechanism of the interaction that gives rise to the inductive effects has not been clarified yet. A recent paper by Nemnes and co-workers^45^ has suggested a classification of recombination effects in terms of either current or charge dominated coupling. In the following we further elaborate on such framework, to explain experimental trends observed in the literature by decisive different properties of the models.

As already commented on in the literature of halide perovskites, there are many observations of inductive features combined with ordinary capacitive features;^16,46−49^ see ref (41) and references therein. The inductive feature becomes very prominent in perovskite memristors as shown in Figure 2b.^22,50^ Therefore, a number of equivalent circuit models including inductors have been developed to account for such features and fit the experimental spectra. A common situation is shown in Figure 3 for a MAPbBr perovskite solar cell. The impedance spectra at low voltages consists of a double arc and it is fully capacitive, Figure 3a. But at a certain applied voltage, a transformation occurs in which the low frequency arc vanishes and gives rise to an inductive arc in the fourth quadrant, Figure 3b–d. This transformation and the associated transition from capacitive to inductive (“inverted”) hysteresis have been explained in detail recently.^17^ The model used to explain the data is shown in Figure 3e, and the resulting time constants are presented in Figure 3f.

**Figure 3:**
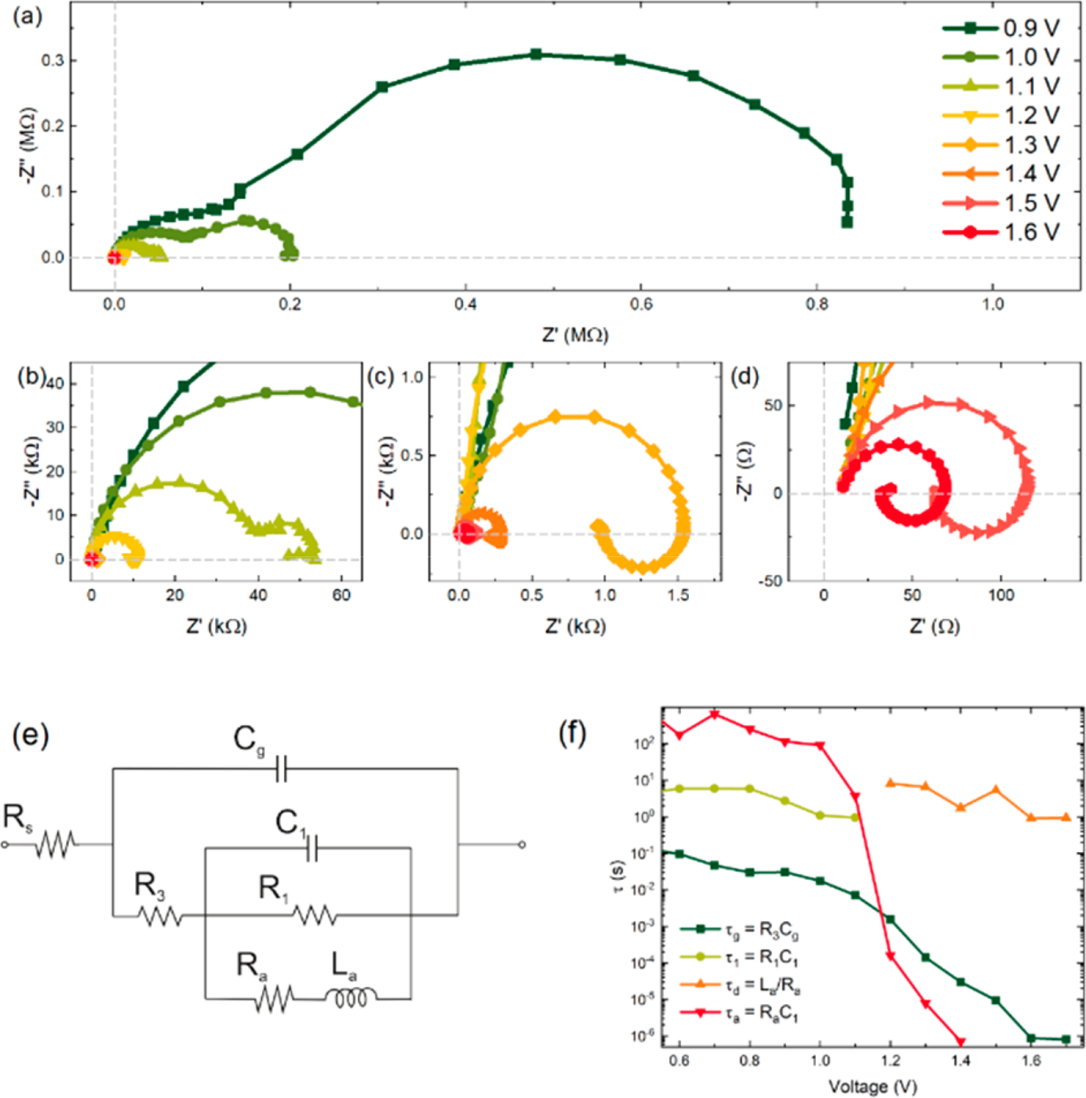
(a–d) Representative IS spectral evolution of a MAPbBr solar cell at *V*_app_ = 0.9 to 1.6 V under dark conditions. (e) Equivalent circuit model. (f) Evolution of different time constants obtained from the impedance parameters as a function of voltage. Reproduced from ref (17). Copyright 2022 American Chemical Society

As mentioned in the introductory remarks, we can distinguish two ways to generate a chemical inductor to account for memory effect in a conducting system. One is to use a current based model of the type of eq 1. Such current-based recombination is developed in ref (17) and provides the (*R*_a_, *L*_a_) line shown in Figure 3e. Note that parallel *R*_1_ and *C*_1_ lines are added in the equivalent circuit model to account for the low frequency arc at low voltages, but these features are not intrinsically related to the inductor. When the data of Figure 3a–d is fitted and the correspondent time constants are calculated, we find an important result. The capacitive and inductive time constants remain approximately constant and continuous across the transformation of capacitor to inductor; see Figure 3f. It is a striking coincidence that such a match occurs if the elements have an independent origin, as it happens in the models in which the slow variable is a recombination current.^17^

Let us discuss the alternative possibility introduced in eq 4. Here the slow variable is a charge *Q*_S_ depending on ionic kinetics that creates two simultaneous effects: surface recombination and surface charging. The first model of this type is presented in ref (11) where a slow surface charge that is a function of an internal voltage is introduced as a surface recombination variable, *Q*_S_ (*V*_S_), according to previous observations of slow photovoltage decays.^51^ This model^11^ generated the equivalent circuit of Figure 1a that we now discuss as a general construction. A description of a halide perovskite memristor^22^ was first modeled with explicit charge feature . Since the capacitor *C*_A_ is in series to the inductor, this line is electrically “blocked” at the electrode and can be associated with an ionic dynamics. The significance of the series combination (*R*_A_, *C*_A_) to account for features observed at intermediate frequencies has been remarked on.^39,52^

By fitting again the data of Figure 3 to the model of Figure 1a, we obtain the results shown in Figure 4. The model provides a good match to the spectra. We remark that the model is able to capture the final turn of the data toward the first quadrant at low frequencies as in Figure 1d, Figure 4f–h. The parameters resulting from the fit are shown in Figure 5. The system can now be described by two essential time constants shown in Figure 5d. The fast time constant τ_m_ follows the variation of *R*_b_, since the geometric capacitance *C*_m_ is a constant. The slow time constant τ_L_ = τ_A_ shows a very weak dependence with the voltage. These features of the main time constants of halide perovskite solar cells have been well described by Garcia-Belmonte and co-workers.^53^ The new finding is that the slow time constant persists to be nearly independent of voltage even when the low frequency capacitance is not observed as it is engulfed by the inductor at high voltage.

**Figure 4:**
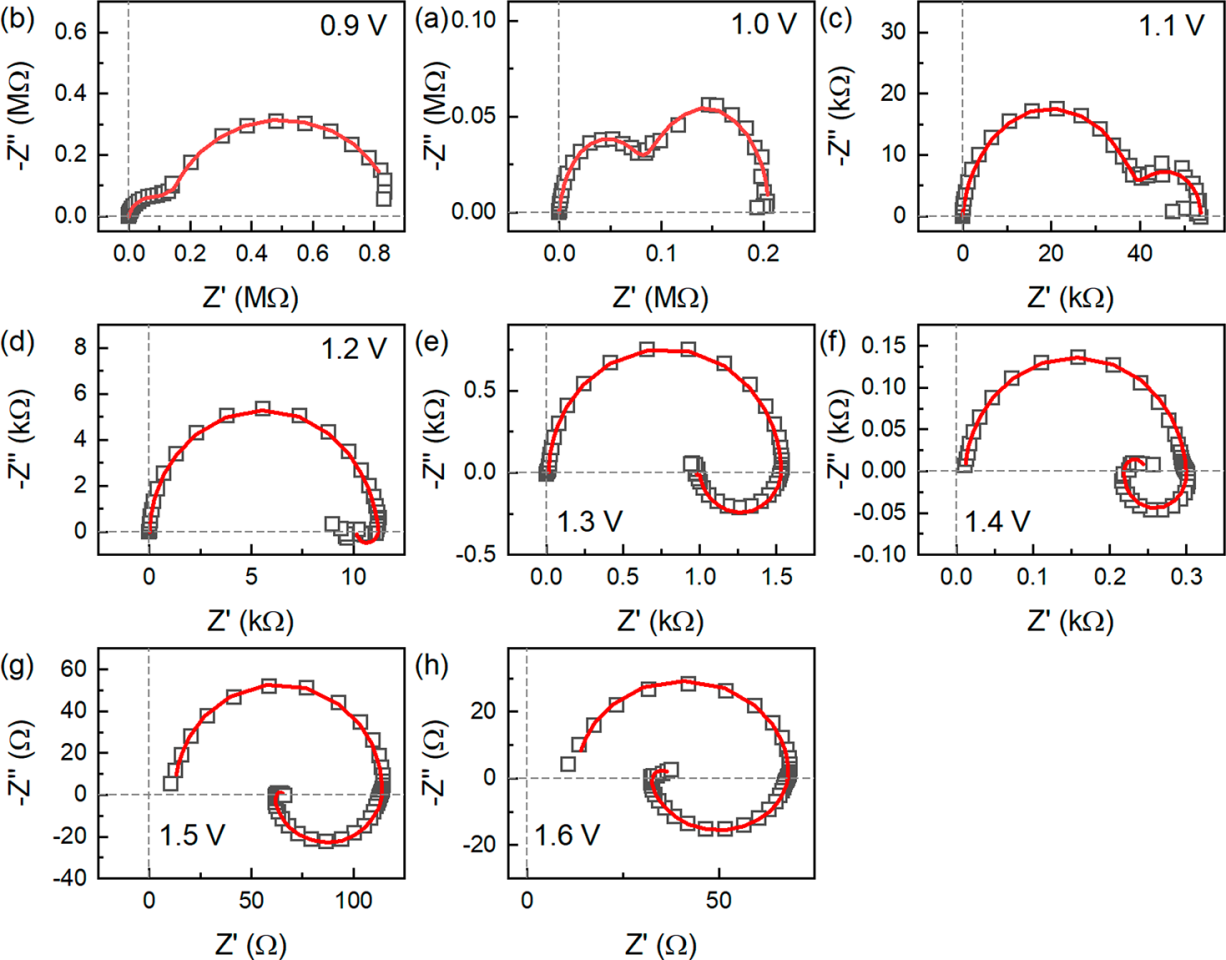
(a–h) Impedance spectra of a MAPbBr solar cell in the dark. Fitting to model of Figure 1a.

**Figure 5:**
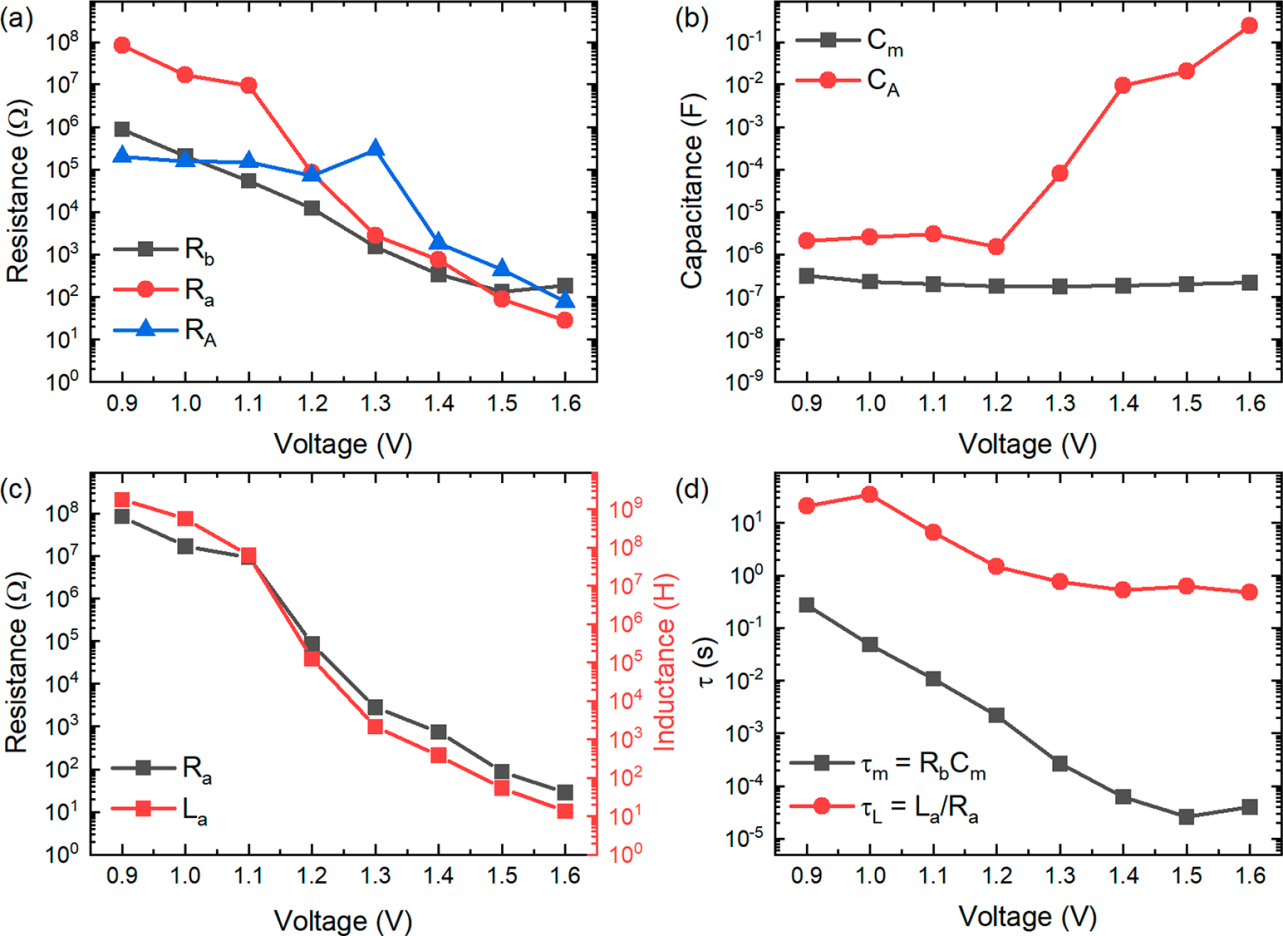
(a–d) Parameters resulting from fit of spectra of a MAPbBr solar cell in the dark to model of Figure 1a.

The charge coupling effect has also been found necessary to describe IS data of illuminated memristors.^38^ A variety of models in the literature can be analyzed in this framework of either charge/current coupling. We refer the reader to ref (45) and references therein. Here we remark that the model based on surface charge immediately explains the observation of continuity of times constants in Figure 3f, by the property discussed in eq 17. We conclude that hysteresis and inductive effects in halide perovskites are associated to a surface recombination charge *C_X_sx̂*, in addition to the current *f_x_x̂*/*R_I_* (eq 6) related to slow ionic effects that increase the surface electronic charge density.^51^

As already indicated previously, we have recently analyzed the general properties of stability of the class of models of eqs 1–2).^21,30,34,35,54^ In these papers we have explained generally that instability, bifurcation, and self-sustained oscillations can be caused by negative resistances of negative inductors, but so far no model contains a truly negative capacitance of the type suggested in ferroelectrics.^19,20,55^

Let us carry out a similar analysis of the class of models of eqs 4–5. The equivalent circuit elements arise from the partial derivatives of the coupling functions *f*, *g* that define the model, by eqs 9–13. We can classify all the signs of the equivalent circuit elements accordingly. The result is shown in Table 1. Note that *R*_b_ is left positive in all cases, since the effect of a negative *R*_b_ is well-known and does not affect the other branches.^25,34^ The table extends the previous result^30^ for the negative inductor, and the main finding is a truly negative capacitance in the combinations observed in the last column.

**Table 1:** Signs and Values of the Circuit Elements and Time Constant

	*f*_*u*_	*g*_*x*_	*g*_*u*_	*f*_*x*_	*f*_*x*_*g*_*u*_	*R*_b_	*R*_a_	*L*_a_	τ_L_	*R*_A_	*C*_A_
						*R*_I_/*f*_u_	–*R*_I_*g*_x_/*f*_x_*g*_u_	*R*_I_τ_k_/*f*_x_*g*_u_	–τ_k_/*g*_x_	τ_k_/*C*_*X*_*g*_u_	–*g*_u_/*g*_x_*C_X_*
1	+	-	+	+	+	+	+	+	+	+	+
2	+	-	+	-	-	+	-	-	+	+	+
3	+	-	-	+	-	+	-	-	+	-	-
4	+	-	-	-	+	+	+	+	+	-	-
5	+	+	+	+	+	+	-	+	-	+	-
6	+	+	+	-	-	+	+	-	-	+	-
7	+	+	-	+	-	+	+	-	-	-	+
8	+	+	-	-	+	+	-	+	-	-	+

It is interesting to speculate on the implications for the time domain techniques. The large scale perturbation techniques in the time domain are connected to the small signal impedance results, but the transformation is not straightforward. This topic has been discussed in refs (56 and 57).

In summary, in this work a general type of dynamical equations is suggested. The traditional neuron-style models for conducting systems with a slow variable have been complemented with an associated capacitive term interpreted as a charging of the slow variable that does not exist in the original neuron models. We find this term very useful to describe ionic-electronic devices as MAPbBr halide perovskite solar cells and memristors in the dark. The reason is that apparently different capacitive and inductive phenomena observed in impedance spectroscopy measurements can be explained by a single unified mechanism. The capacitive coupling also produces the possibility of an intrinsic negative capacitance distinct from the typical inductive effects.

## Supporting Information Available

Transparent Peer Review report available (PDF)
