# Supplementary material for: Electrical Charge Coupling Dominates the Hysteresis Effect of Halide Perovskite Devices
Source: J Phys Chem Lett. 2023 Jan 24;14(4):1014–21. doi: 10.1021/acs.jpclett.2c03812 (PMC10883608; doi:10.1021/acs.jpclett.2c03812)
Supplement: Supplementary file 1 — jz2c03812_si_001.pdf [file jz2c03812_si_001.pdf]

jz-2022-038128.R1

Name: Peer Review Information for "Electrical Charge Coupling Dominates the Hysteresis Effect of Halide Perovskite Devices"

First Round of Reviewer Comments

Reviewer: 1

Comments to the Author

The author proposes a model explaining halide perovskite devices' complex and dynamic behaviour. The model extends on known models of biological neurons and can explain the transition from the capacitor to inductor-like behaviour often observed in impedance spectroscopy of halide perovskites. The author finds that the timescales for capacitive and inductive effects are coupled, indicating that they originate from a single unified mechanism - which is an important finding. The results will be significant for moving forward using halide perovskites for resistive switches and memristors. I suggest the paper be published after minor revisions, as stated below.

The author compares impedance spectra obtained by the model with measured ones from the literature. To validate the applicability of the proposed model, the author should fit the data with the proposed circuit, show the fit results, and quantitatively state the fit results.

The author shows that the model explains halide perovskites' complex and dynamic behaviour in the frequency domain. Is the same true for the time domain?

I suggest adding units to the formula or the variables to allow the reader to follow equations easily.

Reviewer: 2

Comments to the Author

The manuscript by Bisquert describes various equivalent circuit and extend the explanation towards neuron-style models that lead to chemical inductor by introducing a capacitive coupling. This is very interesting.

I have following suggestion or clarification to improve the manuscript.

Fig. 1 explain various circuit, while Fig.2 and Fig. 3 are explanation based on published results and credit and ref. are given. Is the current article is a "research article" or "perspective" or "commentary"? Accordingly the text and abstract, and conclusion should be presented.

The presented model's are an attempt to unify the impedance response of perovskite material (MAPbBr). With the author extended model they explain naturally previous observations concerning the transition from capacitor to inductor in impedance spectroscopy up to 1.6V.

The text and title reflect persisting hysteresis effects in perovskites- but the analysis presented is in dark conditions, this does not truly support material behaviour, which are know to behave differently under light and heat condition. These claims can be verified better under light condition for accuracy and validation of results

I will also suggest to modify the Abstract, and focus on MAPbBr as perovskites, as the text for the "development of switching memory devices that can be used in information storage and brain-like computation" is not supported in this work.

Since it is a single author article, I will also suggest to replace "we" with other word in conclusion section "In summary, we have complemented".

Author's Response to Peer Review Comments:

Journal: The Journal of Physical Chemistry Letters
Manuscript ID: jz-2022-038128
Original Submission Date: 15-Dec-2022
Title: "Electrical Charge Coupling Dominates the Hysteresis Effect of Halide Perovskite Devices"
Author(s): Bisquert, Juan

Reviewer(s)' Comments to Author:

Reviewer: 1

Recommendation: This paper is publishable subject to minor revisions noted. Further review is not needed.

Comments:
The author proposes a model explaining halide perovskite devices' complex and dynamic behaviour. The model extends on known models of biological neurons and can explain the transition from the capacitor to inductor-like behaviour often observed in impedance spectroscopy of halide perovskites. The author finds that the timescales for capacitive and inductive effects are coupled, indicating that they originate from a single unified mechanism - which is an important finding. The results will be significant for moving forward using halide perovskites for resistive switches and memristors. I suggest the paper be published after minor revisions, as stated below.

I thank the reviewer for the positive comments

The author compares impedance spectra obtained by the model with measured ones from the literature. To validate the applicability of the proposed model, the author should fit the data with the proposed circuit, show the fit results, and quantitatively state the fit results.

Thanks for the suggestion. The data have been fitted and are shown as the new Fig. 4 and 5.

The author shows that the model explains halide perovskites' complex and dynamic behaviour in the frequency domain. Is the same true for the time domain?

The large scale perturbation techniques in the time domain are connected to the impedance results but the transformation is not straightforward. This topic has been discussed in Refs. [1,2]. I have commented this point in the paper.

I suggest adding units to the formula or the variables to allow the reader to follow equations easily.

I have added after Eq. (5): Note that units of x and C_m must be adjusted so that I is in amperes, u in volts, C_m in farads, and t, τ_k in seconds.
In Fig. 1: Z' and Z'' are in units of Ω.

Reviewer: 2

Recommendation: This paper is probably publishable, but major revision is needed; I do not need to see future revisions.

Comments:
The manuscript by Bisquert describes various equivalent circuit and extend the explanation towards neuron-style models that lead to chemical inductor by introducing a capacitive coupling. This is very interesting.

Thank you for the positive comments.

I have following suggestion or clarification to improve the manuscript.
Fig. 1 explain various circuit, while Fig.2 and Fig. 3 are explanation based on published results and credit and ref. are given. Is the current article is a "research article" or "perspective" or "commentary"? Accordingly the text and abstract, and conclusion should be presented.

The paper introduces a new class of dynamical equations, Eqs. (4) (5). This is the new scientific contribution. Therefore I think this is an ordinary Letter paper.

The presented model's are an attempt to unify the impedance response of perovskite material (MAPbBr). With the author extended model they explain naturally previous observations concerning the transition from capacitor to inductor in impedance spectroscopy up to 1.6V.
The text and title reflect persisting hysteresis effects in perovskites- but the analysis presented is in dark conditions, this does not truly support material behaviour, which are know to behave differently under light and heat condition. These claims can be verified better under light condition for accuracy and validation of results

The paper extracts conclusions from two previous papers[3,4]. These studies have been made in the dark because they enable to relate the behaviour of solar cells and memristors.

It is remarkable that the MAPbBr memristor[4] requires the capacitive coupling that is suggested in Eq. (4). The study under illumination is certainly interesting but the hysteresis in the dark is already huge and enables to draw conclusions.

I will also suggest to modify the Abstract, and focus on MAPbBr as perovskites, as the text for the "development of switching memory devices that can be used in information storage and brain-like computation" is not supported in this work.

As mentioned earlier, MAPbBr has been analyzed as a solar cells, and also as a switching memory. The equations (4) and (5) suggested in this work refer to a broad class of dynamical behaviours. We have clarified this point in the abstract and in the conclusions to the paper.

Since it is a single author article, I will also suggest to replace "we" with other word in conclusion section "In summary, we have complemented".

We express the conclusion as follows:

In summary, in this work a general type of dynamical equations is suggested. The traditional neuron-style models for conducting systems with a slow variable, has been complemented with an associated capacitive term that does not exist in the original neuron models. We find this term very useful to describe ionic-electronic devices as MAPbBr halide perovskite solar cells and memristors in the dark.

(1) Bisquert, J.; Guerrero, A.; Gonzales, C. Theory of Hysteresis in Halide Perovskites by Integration of the Equivalent Circuit, *ACS Phys. Chem Au* **2021**, *1*, 25-44.

(2) Bisquert, J. Interpretation of the Recombination Lifetime in Halide Perovskite Devices by Correlated Techniques, *J. Phys. Chem. Lett.* **2022**, *13*, 7320–7335.

(3) Gonzales, C.; Guerrero, A.; Bisquert, J. Transition from capacitive to inductive hysteresis: A neuron-style model to correlate I-V curves to impedances of metal halide perovskites, *J. Phys. Chem. C* **2022**, *126*, 13560–13578.

(4) Munoz-Diaz, L.; Rosa, A. J.; Bou, A.; Sanchez, R. S.; Romero, B.; John, R. A.; Kovalenko, M. V.; Guerrero, A.; Bisquert, J. Inductive and Capacitive Hysteresis of Halide Perovskite Solar Cells and Memristors Under Illumination, *Frontiers in Energy Research* **2022**, *10*, 914115.
